# Clinical characteristics, management, and outcomes of diseases caused by mercury overexposure: a systematic review of case reports and case series

**DOI:** 10.3389/fpubh.2026.1750332

**Published:** 2026-02-02

**Authors:** Yun Shi, Minyan Wang, Haojie Ni, Sisi Lin, Dieyu Ma, Zhizhen Zhou, Conghua Ji

**Affiliations:** School of Public Health, Zhejiang Chinese Medical University, Hangzhou, China

**Keywords:** case report, disease, mercury, review, treatment

## Abstract

**Background:**

Mercury poisoning remains a serious public health issue due to its multiple exposure routes and diverse clinical presentations. However, existing clinical evidence is fragmented, especially regarding real-world case data. This study addresses the gap by systematically reviewing global case reports to analyze clinical features, diagnosis, and treatments, thereby offering more robust evidence for clinical practice.

**Methods:**

Case reports of diseases caused by accidental mercury exposure, published from January 1950 to April 2025, were identified through a comprehensive search of three electronic databases: PubMed, Embase, and Web of Science. Following quality assessment using the Joanna Briggs Institute tool, data extraction was conducted on demographic characteristics, clinical manifestations, diagnostic methods, exposure sources, treatment modalities, and patient outcomes to facilitate further analysis.

**Results:**

This study analyzed 80 articles encompassing 126 cases of mercury poisoning, involving 61 males, 60 females, and 5 cases of unspecified sex. Patient ages ranged from 45-day-old neonates to 88-year-old adults. Clinical manifestations were diverse, primarily featuring systemic, respiratory, neurological, and gastrointestinal symptoms. Domestic/environmental exposure was the most common poisoning route (59.5%), followed by medical/iatrogenic exposure (16.7%) and occupational exposure (13.5%). Elemental and inorganic mercury were the predominant forms involved. Treatment primarily included chelation therapy, supportive care, and pharmacological interventions. Chelating agents were administered to 84.1% of patients, with DMSA, Dimercaprol (BAL), DMPS, and D-penicillamine being the most frequently used. Outcomes included complete recovery in 48.4% of cases, death in 13.5%, and long-term sequelae in some patients.

**Conclusion:**

Mercury poisoning induces severe and multisystemic symptoms, notably neurological damage. Public awareness remains insufficient, and universally accepted diagnostic criteria are still lacking. This study highlights the urgent need to enhance public education, refine clinical guidance, and advance research toward standardized management protocols.

**Systematic review registration:**

CRD420251133420.

## Introduction

All humans are exposed to some level of mercury, which has been historically used in medicine, agriculture, and industry. According to the World Health Organization, mercury has been ranked as one of the 10 most hazardous substances in the world (1). Whether through contaminated food chains, occupational exposure, or environmental pollution, mercury exposure can cause serious and irreversible health damage (2). Particularly hazardous is methylmercury, which bioaccumulates in aquatic ecosystems, making consumption of contaminated fish the primary human exposure pathway (3). Chronic low-dose exposure can lead to irreversible neurological damage in adults and impaired fetal neurodevelopment (4, 5), whereas acute high-dose exposure manifests differently depending on the route. For instance, inhalation of elemental mercury vapor primarily targets the respiratory system, causing severe pneumonitis, while ingestion of inorganic mercury salts causes corrosive damage to the gastrointestinal tract (6). Mercury’s unique ability to cross biological barriers, including the blood–brain and placental barriers, enables it to extensively disrupt cellular functions across multiple organ systems (7). Despite international regulatory measures, ongoing industrial emissions, improper disposal of mercury-containing products, and consumption of contaminated seafood continue to drive the occurrence of mercury-related diseases. The persistent emergence of both chronic poisoning cases and acute exposure incidents underscores the urgent need to refine prevention strategies and optimize treatment protocols to address this enduring environmental health challenge.

Current clinical research on mercury poisoning shows an uneven distribution of evidence (8). While epidemiological studies provide macro-level data on population exposure and experimental research elucidates some toxicological mechanisms, case reports and case series that best reflect real-world clinical management remain systematically unintegrated. Although methodologically limited, these studies offer invaluable clinical details beyond textbooks, particularly in documenting rare poisoning scenarios, atypical clinical presentations, and individualized treatment responses. However, significant variations exist among these scattered cases in diagnostic criteria, treatment protocols and outcome assessments, making it challenging for clinicians to extract universally applicable conclusions.

This study aims to address this research gap through a systematic review of globally published case reports on mercury poisoning. We will focus on analyzing clinical manifestations, diagnostic methods and the effectiveness of treatment strategies, while examining how exposure routes, mercury species and population characteristics affect outcomes. By synthesizing these primary clinical data, this review expects to provide more reliable evidence for clinical practice and identify directions for future research.

## Methods

This systematic review aimed to gather and evaluate the key characteristics of mercury overexposure-related diseases case reports and series published in the literature. The literature search followed the Preferred Reporting Item for Systematic Reviews and Meta-Analyses (PRISMA) guidelines (9). The study protocol was registered on PROSPERO (registration number, CRD420251133420).

### Search strategy

The literature search was conducted in April 2025 across three electronic databases: PubMed, Embase, and Web of Science. The search scope included all publications from January 1950 onwards to capture the entirety of the modern evidence base on the topic.

In PubMed, we employed a targeted search strategy using both MeSH terms and title/abstract keywords. The search strategy incorporated Boolean operators (AND, OR) to systematically combine key concepts while maintaining search sensitivity. The search string included: (“Mercury”[Mesh] OR “Methylmercury”[Title/Abstract] OR “Mercury Poisoning”[Title/Abstract] OR “mercuric”[Title/Abstract] OR “Mercury poisoning”[Title/Abstract] OR “Mercury toxicity”[Title/Abstract]) combined with (“Case report”[Title/Abstract] OR “Case series”[Title/Abstract]). Similar search term combinations were adapted for Embase and Web of Science to account for differences in each database’s indexing system and search syntax. All identified records from the three databases were exported to EndNote reference management software.

### Inclusion and exclusion criteria

The study selection followed predetermined eligibility criteria. We included peer-reviewed case reports and case series that provided comprehensive documentation of therapeutic interventions for mercury poisoning. Only English-language publications with accessible full texts were considered. We excluded studies that: (1) reported mixed heavy metal exposures without isolated mercury poisoning; (2) described cases of deliberate mercury ingestion; (3) were unavailable in full-text format; or (4) lacked essential clinical details regarding treatment regimens. This selective approach ensured that we focused specifically on unintentional mercury poisoning cases with well-documented management strategies.

### Data extraction

Two authors (Y. S. and M. W.) independently screened titles and abstracts after removing duplicates and assessed all relevant full-text articles. Disagreements between the observers were resolved by discussion or, if necessary, by a third author (H. N.) adjudication.

The two authors independently extracted the following study characteristics and descriptive data of the included patients, as well as outcome data, using a standardized form: gender, age, chief complaint, complications, exposure source, mercury species, mercury concentration, diagnostic criteria, treatment modalities, treatment efficacy, and outcomes of the mercury poisoning cases.

### Quality assessment

The authors independently assessed the quality of the evidence using the JBI (Joanna Briggs Institute) Critical Appraisal Checklist for case reports (10). The checklist contains eight items, and encompasses comprehensive evaluation of patient demographics, chronological medical history documentation, baseline clinical presentation characteristics, diagnostic methodology and findings, therapeutic interventions, post-intervention clinical progression, recorded adverse events, and derived clinical insights.

## Results

The initial search identified 4,585 records. PubMed: 148 articles, Embase: 1984 articles, and web of science: 2453 articles.

**Figure 1:**
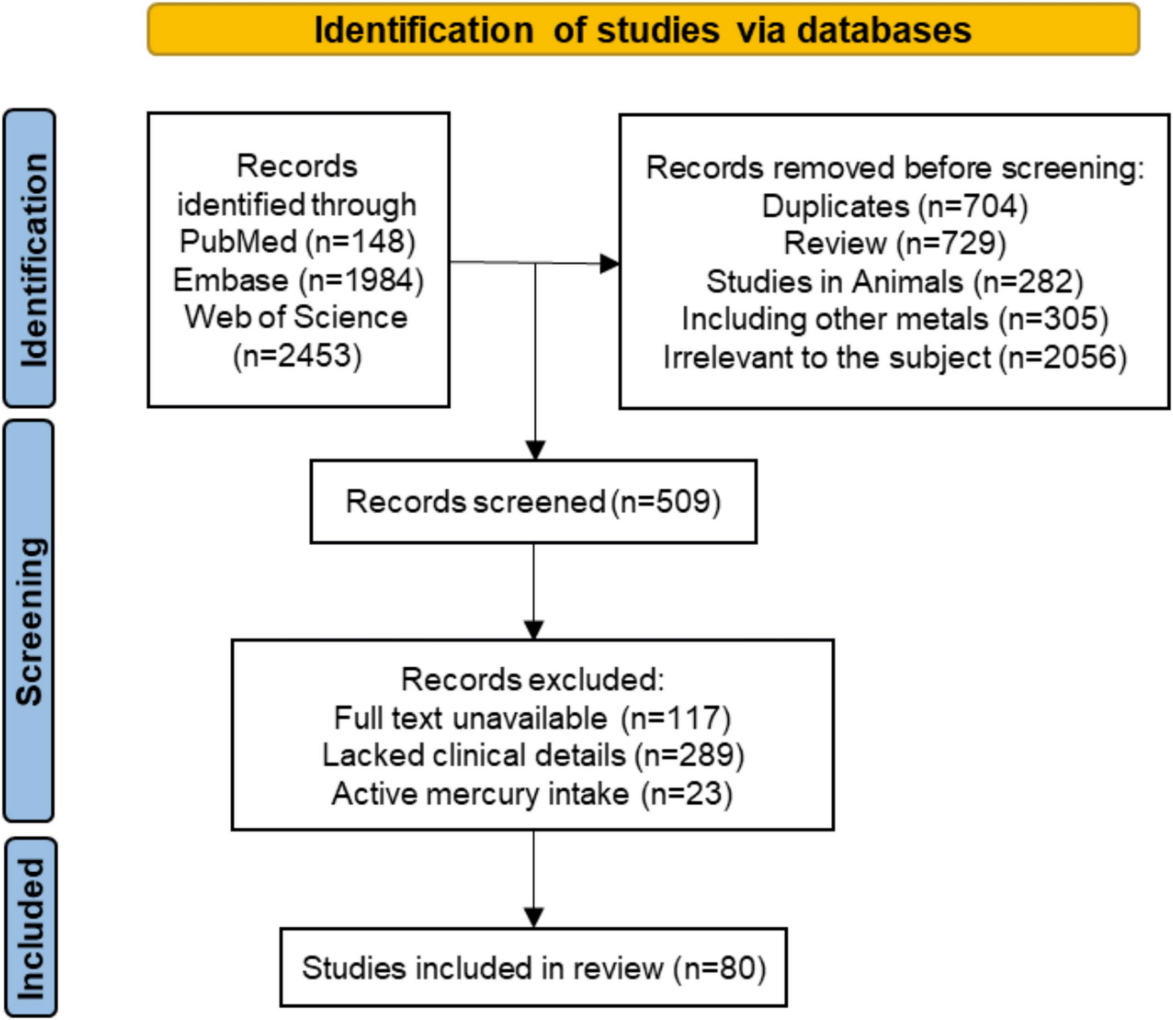
Review search strategy.

After removing 704 duplicates, 3,881 records were screened based on their title and abstract. Of these, 509 studies were sought for retrieval. Finally, 80 articles were included in this study (11–90). The PRISMA diagram (91) depicting search results details and reasons for exclusion are provided in Figure 1. Full details and data extracted from each eligible paper can be found in Supplementary Table 1.

### Studies overview

The included studies spanned a period from 1957 to 2025, covering nearly 70 years of research. Among them, 60 were single-case reports of mercury poisoning (11, 13, 15–17, 20, 22–29, 33, 35, 37–41, 43, 44, 46–52, 54, 56, 57, 59, 60, 62–69, 71–80, 83–89), while 20 were case series involving multiple patients (12, 14, 18, 19, 21, 30–32, 34, 36, 42, 45, 53, 55, 58, 61, 70, 81, 82, 90). Geographically, the United States contributed the most studies (24 articles, 30.0%) (12–14, 16, 18, 22, 24, 27, 29, 31, 32, 34–36, 42, 43, 56, 57, 59, 72, 77, 79, 80, 82), followed by China (11 articles, 13.8%) (41, 46, 49, 65, 68, 73, 81, 85–87, 90) and Turkey (9 articles, 11.3%) (45, 50, 51, 53, 55, 58, 61, 64, 67).

Among the 80 included studies, 29 (36.3%) provided complete responses to all eight questions in the JBI checklist. All articles offered detailed descriptions of the patient’s clinical condition and summarized valuable lessons from the case. However, only 36 studies (45.0%) identified and described adverse events (harms) or unanticipated events. Overall, the included case reports demonstrated satisfactory completeness (Supplementary Table 2).

These 80 studies collectively reported 126 mercury poisoning cases. Gender was documented in 121 cases (96.0%), comprising 61 males and 60 females. The gender of the remaining 5 cases (4.0%), all of whom were pediatric patients, was not reported. The age range of patients was remarkably broad, from 45-day-old neonates to 88-year-old individuals.

Analysis of mercury exposure sources revealed domestic/environmental exposure as the primary route (75 cases, 59.5%), followed by medical/iatrogenic exposure (21 cases, 16.7%) and occupational exposure (17 cases, 13.5%). Regarding treatment, among the 126 patients, 106 (84.1%) received chelation therapy, while the remaining 20 (15.9%) received supportive care only. Among the 106 patients who received chelation therapy, monotherapy with a single agent was the predominant strategy (85 patients, 80.2%). The most frequently used agents in monotherapy were DMSA (dimercaptosuccinic acid, *n* = 26), dimercaprol (BAL, *n* = 19), D-penicillamine (*n* = 18), and DMPS (sodium 2,3-dimercaptopropane-1-sulfonate, *n* = 18). Combination or sequential regimens involving more than one chelator were employed in 17 patients (16.0%). For the remaining 4 patients (3.8%), the specific chelating agents were not documented.

In terms of clinical outcomes, 61 patients (48.4%) achieved complete recovery, while 17 cases (13.5%) resulted in mortality. With the exception of eight patients who were lost to follow-up, the remaining cases experienced sequelae or had a poor prognosis (31.7%).

### Demographic characteristics

Among the 126 mercury poisoning cases included in this study, 123 (97.6%) had complete age documentation. Analysis of this subset revealed that adults (18–59 years) constituted the largest group (62 cases, 50.4%), followed by pediatric patients (<18 years, 55 cases, 44.7%). Within the pediatric subgroup, nearly half were infants under 3 years old (25 cases, 45.5%). Geriatric patients (>60 years) represented the smallest proportion (6 cases, 4.9%). The remaining 3 cases lacked age data.

Gender was recorded in 121 cases (96.0%), showing balanced distribution (male: female = 61:60, ratio 1.02:1).

### Clinical presentation and diagnosis

**Figure 2:**
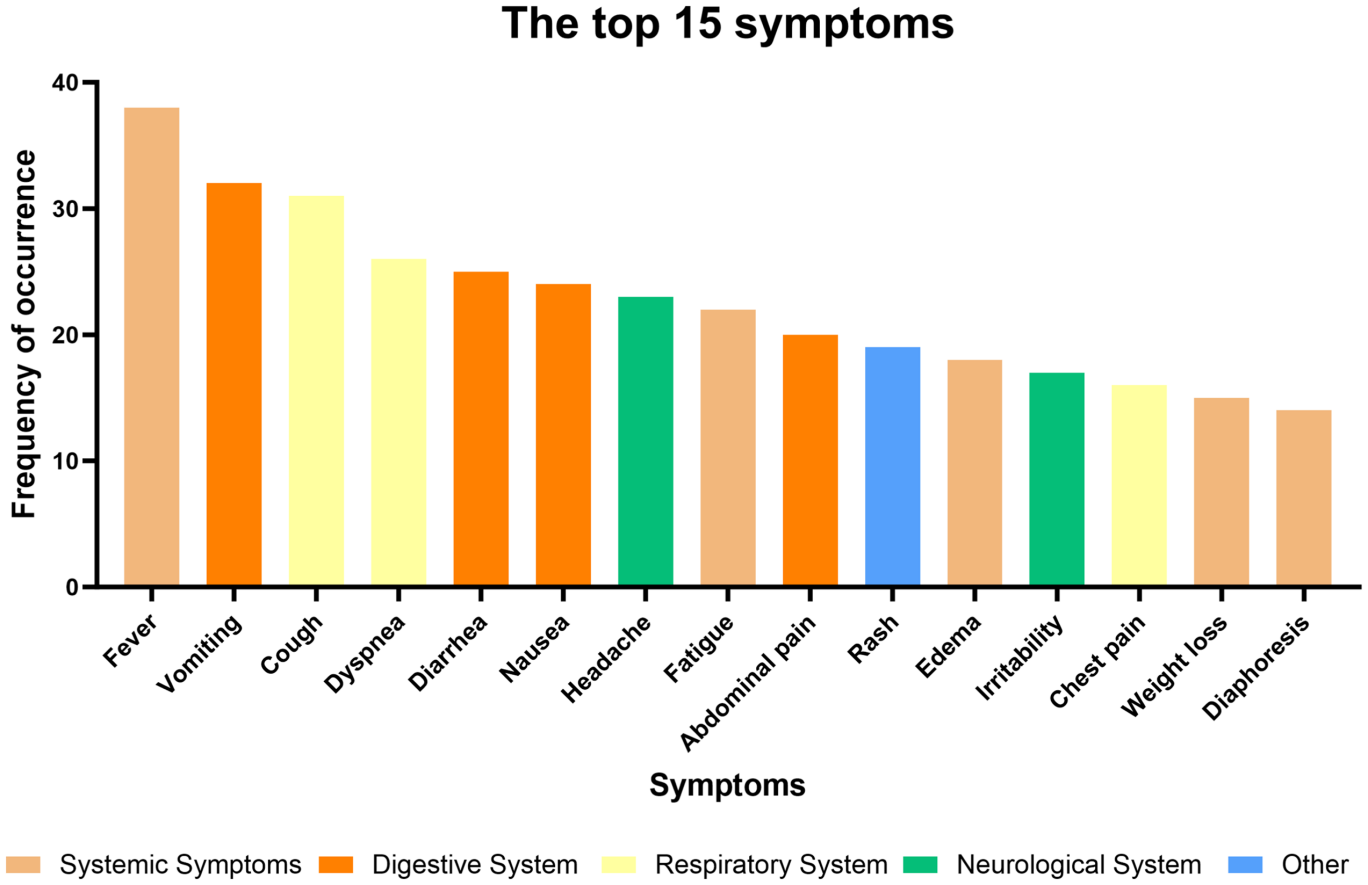
The top 15 most frequent symptoms in 122 patients.

The clinical manifestations of all 126 patients included in this study were thoroughly documented. Analysis revealed significant heterogeneity in the symptom profiles among different patients. However, individuals from the same exposure event exhibited highly similar clinical presentations, indicating a strong association between specific exposure sources and distinct symptom patterns. As shown in Table 1, symptoms were categorized by organ system, primarily including constitutional, respiratory, neurological, gastrointestinal, urinary, circulatory, and dermatological symptoms. At the systemic level, neurological involvement was the most prevalent and consequential category of clinical manifestations. Symptoms within this category were notably complex and diverse, representing the greatest threat to long-term prognosis. At the level of individual symptoms, however, non-specific systemic signs such as fever and vomiting were most frequently documented (Figure 2), often serving as the initial presentation. During diagnosis, these nonspecific symptoms require careful differentiation from similar manifestations caused by other etiologies.

**Table 1:** Classification of major mercury poisoning symptoms.

Classification	Frequent symptoms
Systemic symptoms	Fever; Diaphoresis; Edema; Weight loss; Fatigue/Malaise; Pain
Respiratory system	Bronchitis; Acute Respiratory Distress Syndrome (ARDS); Chemical pneumonitis/Severe lung injury; Pharyngitis/Laryngitis; Hemoptysis; Frequent epistaxis; Respiratory symptoms (cough, shortness of breath, etc.); Mild hypoxemia
Neurological system	Encephalopathy; Headache; Somnolence; Disorientation; Alternating confusion and aggressive behavior; Hallucinations, Delusions, Confabulation; Paraphasia, Dysarthria; Language function deterioration; Recent memory decline; Agraphia; Opisthotonos; Tremor; Choreiform movements; Motor tics, Vocal tics; Instability when standing or walking, Gait instability, Limb incoordination; Progressive paresthesia (numbness in palms, limbs, lower extremities, oral cavity); Neuropathy; Visual acuity decline, Hearing decline; Depression, Irritability, Anxiety, Occasional euphoria; Aggressive behavior; Abnormal posturing during sleep and vivid dreams; Behavioral changes, Bizarre behavior
Digestive system	Nausea, Vomiting; Coffee-ground emesis, Hematemesis (Vomiting blood); Oral lesions (Buccal pain, Oral ulcers, Gingival pain, Metallic taste); Hepatitis/Liver injury; Jaundice, Hepatosplenomegaly; Abnormal liver function tests (LFTs); Diarrhea, Bloody diarrhea (Hematochezia); Bloody stool, Melena (Black, tarry stool)
Urinary system	Acute kidney injury (AKI)/Renal failure; Anuria; Hematuria, Urinary frequency; Foamy urine (proteinuria); Left lumbar region discomfort; Nephritis/Nephrotic syndrome
Circulatory system	Blood pressure abnormalities (Hypertension, Hypotension) Heart rhythm abnormalities (Tachycardia, Palpitations)
Other	Rash, Generalized erythema; Facial and hand lesions, Blisters/Ulcers; Facial pigmentation; Palmar-plantar desquamation; Acrodynia; Intense nasal burning sensation, Nasal pain; Chronic otitis media

The diagnosis of mercury poisoning was primarily confirmed by measuring blood and/or urinary mercury levels. Among the cases, 108 (85.7%) had clearly documented blood and/or urinary mercury data, while less cases provided hair and nail mercury values (21). However, substantial variation was observed in the medical reference ranges used across different cases, resulting in a lack of uniform criteria for interpretation. Diagnosis, therefore, largely relied on observed levels significantly exceeding the reference values provided in each respective context. The remaining cases (12.7%), which lacked mercury biomonitoring data, were predominantly reported in literature prior to the year 2000. In these instances, diagnosis was based on a clear history of mercury exposure supported by consistent clinical symptoms.

Among cases with quantitative biomonitoring data, mercury concentrations exhibited considerable heterogeneity, spanning from mildly elevated to life-threatening extremes. Severe elevations were consistently associated with fatal outcomes. A predominant pattern emerged in which urinary mercury levels substantially exceeded concurrent blood mercury levels. In contrast, a distinct minority of cases presented with the inverse profile, characterized by markedly elevated blood mercury concentrations alongside relatively low urinary levels. Furthermore, several cases documented extremely elevated mercury concentrations in hair and nail samples.

### Mode of exposure

A statistical analysis of exposure sources was conducted on 123 patients with clearly documented mercury exposure. The primary sources were categorized into five groups: medical/iatrogenic exposure, occupational exposure, domestic/environmental exposure, cosmetic exposure, and folk remedy exposure (Table 2). Among these, domestic/environmental exposure was the most frequently observed. Enclosed spaces, combined with either intentional or accidental contact with mercury and improper handling methods, often lead to mercury poisoning. Such incidents frequently result in multiple cases of poisoning within a household, representing a significant public health concern. Exposure from mercury-containing preparations also constituted a considerable proportion. Medical/iatrogenic exposure, which includes historical or regulated uses within traditional medicine systems as well as adulterated products, was documented in 21 cases (16.7%). In contrast, folk remedy exposure, defined as the use of mercury based on advice from unlicensed practitioners or folk beliefs without medical basis, was identified in 2 cases (1.6%). Cosmetic exposure from illegal skin-lightening products was reported in 8 cases.

**Table 2:** Main sources of mercury exposure mentioned.

Exposure category	Specific source	Typical risk behavior
1. Medical/Iatrogenic Exposure		
Pharmaceutical	Mercury-containing diuretics (Mersalyl)	Historical use for conditions like heart failure.
	Topical antiseptics (ammoniated mercury ointment)	Treatment of skin diseases/lightening (not recommended).
	Mercury-containing solutions (mercuric chloride)	Surgical irrigation or wound treatment.
	Preservatives (Thimerosal)	Middle ear irrigation.
Traditional/Herbal Preparations	Mercury-containing traditional medicines	Dermal and long-term use.
Medical Devices	Mercury thermometers, sphygmomanometers	Breakage leading to spillage or accidental injection.
2. Occupational Exposure		
Dentistry	Dental amalgam handling	Hand contamination; smoking in contaminated clinics.
Mining, Metallurgy and Gilding	Heating mercury-gold/silver amalgam	Refining precious metals without protection.
	Recovery of metals	Exposure to mercury vapor in high-temperature workshops.
Chemical and Laboratory Work	Handling metallic mercury; chemical explosions	Accidents during production or experimentation.
Specific Industries	Ship anti-fouling paint, Battery production/recycling	Manufacturing or recycling processes.
3. Domestic/ Environmental Exposure		
Domestic Ore Processing	Heating mercury amalgams at home	Refining gold/silver in confined, poorly ventilated spaces.
Elemental Mercury Spills	Mercury brought from schools/labs; broken household items	Spills on floors/carpets; improper cleanup.
Cultural Use	Use of liquid mercury in religious or folk practices	Use in confined spaces, sometimes involving heating.
Historical Contamination	Residing in homes contaminated by previous occupants	Chronic, unknown exposure to residual mercury vapor.
Accidental Ingestion/Injection	Accidental ingestion of mercuric chloride powders; subcutaneous injection from broken devices	Misidentification or improper storage of toxic substances.
Contaminated Food	Consumption of methylmercury-contaminated pork	Point source environmental contamination entering food chain.
4. Cosmetics Exposure		
	Skin-lightening creams, soaps, lotions	Purchased online or illegally; used for months to years.
5. Folk Remedy Exposure		
	Oral remedies for skin diseases	Prescribed by unlicensed practitioners for conditions like psoriasis or eczema.
	Topical ointments for various ailments	Unregulated application based on folk traditions.

Mercury intoxication can result from exposure to all three forms of mercury: elemental, inorganic, and organic (92). Among the cases reviewed, elemental mercury (particularly as mercury vapor) was the most prevalent form, accounting for 84 out of 123 cases. Inorganic mercury was the second most common, observed in 33 cases. In contrast, organic mercury represented a notably small proportion of cases.

Following exposure to elemental mercury, the most prominent manifestation is damage to the nervous system, including finger tremor, severe emotional volatility, memory decline, insomnia, visual impairment, and even ataxia, walking difficulties, deterioration of handwriting, and hallucinations. The respiratory system is acutely impaired after inhalation, causing sudden respiratory distress, cough, dyspnea, and can rapidly progress to acute respiratory distress syndrome. Simultaneously, patients commonly exhibit significant oral paresthesia, salivation, gingivostomatitis, as well as systemic fatigue, weight loss, profuse sweating, and muscle pain.

In patients with inorganic mercury poisoning, gastrointestinal manifestations are significant, including severe vomiting, diarrhea, hematemesis, coffee-ground vomitus, bloody stools, and tarry stools, accompanied by swelling, ulcers, and blisters in the oral cavity and lips. Kidney damage presents as anuria, oliguria, hematuria, frothy urine, and edema progressing from the lower limbs to the entire body. The effects on the nervous system are diverse and notable, showing progressively worsening sensory abnormalities, limb weakness, unsteady gait, muscle fasciculations, insomnia, mood swings, depression, anxiety, and even the appearance of tics, choreiform movements, and ataxia. Furthermore, extreme fatigue, weight loss, skin itching, palpitations, and blood pressure fluctuations are commonly observed systemically.

Organic mercury poisoning primarily induces severe damage centered on the nervous system, presenting as visual impairment, impaired cerebellar function, sensory abnormalities, and slurred speech, severely progressing to quadriplegia and persistent vegetative state, while also accompanied by systemic effects such as renal failure, gastrointestinal symptoms, and oral ulcers.

### Treatment and outcome

The management of mercury poisoning, as evidenced by the case reports in this study, primarily involved the following key aspects: chelation therapy, supportive and symptomatic care, pharmacological management, local treatment, and other interventions (Table 3). The cornerstone of management is the immediate cessation of exposure and elimination of the source. Chelation therapy serves as the central, specific pharmacological intervention for reducing the body’s mercury burden. Adjunctive treatments are then tailored to the patient’s specific symptoms and complications. Overall, the therapeutic strategy is highly individualized and depends on the form of mercury, the route and duration of exposure, the dose, and the patient’s clinical manifestations.

**Table 3:** Main treatment modality.

Treatment modality	Example
Chelation therapy	Dimercaprol (BAL); D-penicillamine; DMSA (Dimercaptosuccinic acid); DMPS (sodium 2,3-dimercaptopropane-1-sulfonate); Edetate calcium disodium (CaNa2EDTA); NAP (N-Acetyl-D-penicillamine); NAC (N-Acetylcysteine)
Supportive and symptomatic care	Oxygen therapy; Mechanical ventilation; High-PEEP Ventilation; Chest tube drainage; Hemodialysis; Peritoneal dialysis; Hemoperfusion; CVVHD (Continuous Veno-Venous Hemodialysis); Intravenous fluids; Electrolyte management; Nutritional support; Tube feeding; Blood transfusion; Albumin infusion; G-CSF (Granulocyte Colony-Stimulating Factor)
Pharmacological management	Corticosteroids; Antibiotics; Antifungals; Antivirals; Diuretics; Antihypertensives; Sedatives; Analgesics; Antihistamines; Antiepileptics; Immunosuppressants; IVIG (Intravenous Immunoglobulin); Antiemetics; Gastric protectants; Activated charcoal; Urine alkalization
Topical therapy	Debridement; Topical antibiotic ointment; Gentian violet; Calamine lotion
Other interventions	Cessation of exposure; Surgical intervention; Environmental intervention; Bronchoalveolar lavage; Erythrocytapheresis

Following a period of treatment, the health status of most patients improved, enabling them to gradually return to normal life, although some individuals with severe mercury poisoning unfortunately died. However, a considerable number of patients were left with long-term sequelae, including respiratory impairments such as pulmonary fibrosis, exertional dyspnea, and reduced diffusing capacity; renal abnormalities such as persistent proteinuria; as well as irreversible neurological damage and cognitive dysfunction. Some patients also developed corneal scarring with vascularization, visual impairment, and recurrent eczema. Pediatric patients, in particular, could exhibit delayed language development. These sequelae significantly impact patients’ long-term quality of life.

## Discussion

### Main findings

Mercury is a common toxic heavy metal. Although its toxicity and associated treatments have been extensively documented in the literature, the overall harm to human health caused by mercury overload as well as the specific circumstances of its treatment still lacks systematic investigation. Therefore, we conducted a PRISMA-compliant systematic review to comprehensively characterize the clinical spectrum and management outcomes of diseases associated with mercury overexposure based on available case reports.

Our work has yielded several key findings. Firstly, we observed that patients with mercury poisoning exhibit prominent systemic symptoms, with high prevalence of manifestations such as fever, sweating, edema, weight loss, fatigue/malaise, and pain. Beyond these, the majority of patients also presented with neurological, gastrointestinal, and respiratory symptoms. Among these, neurological symptoms were the most frequently reported, displayed considerable diversity, were often debilitating, and prone to leaving sequelae. This symptomatic pattern remained consistent across various exposure sources.

Secondly, a significant historical shift in exposure sources was noted. While historically, mercury poisoning was often linked to occupational or medical sources, the cases in this review highlight the growing relative importance of domestic exposures, adulterated consumer products, and hazardous traditional practices (93).

Thirdly, our review delineates a clear therapeutic framework centered on a three-step approach: immediate cessation of exposure, prompt administration of chelating agents, and comprehensive supportive and symptomatic care. The selection of specific chelators and supportive measures was highly individualized, contingent upon the form of mercury and the patient’s clinical status.

Lastly, despite heterogeneity in the reporting standards of the included case reports, our synthesis successfully establishes a foundational clinical profile of mercury poisoning and provides a structured overview of treatment strategies grounded in real-world evidence.

### Interpretation

Mercury poisoning can occur in individuals of any gender, age, or occupation, indicating no specific population is exempt from the risk. Most cases of accidental mercury poisoning included in this study resulted from a lack of awareness regarding the hazards of mercury-containing products, leading to excessive mercury intake without adequate protection. Over time, the primary sources of mercury exposure have shifted: earlier cases were largely associated with medical interventions, whereas recent years have seen a growing incidence of exposure in household and environmental contexts. This trend underscores the importance of public attention to environmental ventilation, the safety of children’s toys, and cosmetic products (94).

The clinical manifestations of mercury poisoning are highly diverse. In addition to systemic symptoms, local signs at the site of exposure are also prominent. This study found that exposure to elemental mercury primarily induced respiratory symptoms, while inorganic mercury exposure more commonly led to local dermatological signs and urinary system impairments. From the effects of different forms of mercury poisoning, we can clearly observe the intrinsic connection between their toxicological characteristics and clinical symptoms. Elemental mercury vapor, due to its high volatility and lipophilicity, is absorbed through the respiratory tract and rapidly crosses the blood–brain barrier, leading to characteristic manifestations centered on damage to the central nervous system (95). In contrast, inorganic mercury salts, owing to their water solubility and corrosiveness, cause direct damage to mucosal tissues upon digestive tract contact, manifesting as severe gastrointestinal inflammation and hemorrhage. They are subsequently excreted via the kidneys, causing renal tubular necrosis and ultimately progressing to acute renal failure. Organic mercury compounds, entering the human body through bioaccumulation, exhibit distinct neuroaffinity and delayed toxicity. Initial symptoms are often subtle but progressively develop into irreversible neurological damage (96). These differences are closely associated with variations in the absorption rates of different mercury forms in the human body.

Assessment of mercury body burden is typically conducted by measuring mercury levels in blood, urine, hair, or nails. Blood mercury concentrations rise rapidly after short-term exposure; however, in cases of chronic exposure, levels may remain elevated even after exposure cessation, reflecting significant bodily accumulation. Urinary mercury concentration, which is stable and easily measured, is commonly used for rapid screening of exposure, though it has limited value in indicating organic mercury burden. Hair mercury concentration serves primarily as a biomarker for long-term methylmercury exposure. Currently, reference values for mercury in these biological matrices lack universal standardization. Based on a synthesis of available literature, this study proposes the following thresholds: blood mercury <10 μg/L (adults) or lower (children), with levels >50 μg/L suggesting poisoning; urinary mercury <20 μg/L or <10 μg/24 h, with levels >50 μg/L indicating overexposure and >100 μg/L warranting therapeutic intervention; hair mercury <1.5 μg/g or 1–2 ppm (97). Following chelation therapy, a sharp transient rise in urinary mercury excretion not only confirms the efficacy of treatment but also substantiates the diagnosis. It is noteworthy that extremely high blood mercury levels are often associated with poor prognosis and may correlate with fatal outcomes. This study emphasizes that poisoning severity is strongly correlated with internal body burden, and both blood and urinary mercury measurements serve as indispensable objective tools for exposure assessment, severity stratification, and guiding treatment. To date, there remains no universally accepted diagnostic criterion for mercury poisoning, and diagnosis continues to rely on a combination biomonitoring and clinical evaluation.

The results demonstrated two predominant biomarker profiles: one characterized by urinary mercury levels substantially exceeding those in blood, and another by the inverse relationship. The observed divergence in biomarker profiles is attributable to fundamental differences in the pharmacokinetics of distinct mercury species (98). Specifically, the prevalent pattern of elevated urinary mercury relative to blood mercury is pathognomonic of exposure to elemental mercury vapor or inorganic mercury compounds. Following absorption, inorganic mercury undergoes renal accumulation and is excreted slowly, resulting in a sustained high urinary concentration that serves as a robust indicator of chronic tissue burden (99). In contrast, the profile dominated by elevated blood mercury with comparatively low urinary excretion is more suggestive of exposure to lipophilic organic mercury, such as methylmercury. This species exhibits a strong affinity for the blood compartment and neural tissues, with a prolonged blood half-life (100) and limited direct renal elimination in its inorganic form (101). Elevated mercury levels in hair and nails primarily reflect integrated, long-term exposure to methylmercury, as it is incorporated into keratin during growth. However, this biomarker is not specific to occupational exposure, which typically involves inorganic mercury or elemental mercury vapor. While such findings provide compelling evidence of systemic accumulation over months, their interpretation in occupational settings requires caution due to the potential for external contamination. Collectively, the substantial heterogeneity in reported reference ranges underscores a significant challenge in cross-study comparisons. This variability emphasizes that clinical diagnosis must integrate both quantitative biomarker levels and a comprehensive assessment of the exposure history and clinical presentation (102).

Treatment strategies primarily include general supportive care, chelating agents, and plasma exchange. Chelation therapy facilitates the complexation and elimination of excess or toxic metals, thereby reducing acute toxicity and mitigating delayed effects. Symptomatic patients require prompt chelation treatment; however, the benefit of chelators in severe poisoning remains uncertain, and indications for such therapy are not fully established (103).

The time course of recovery was inconsistently reported. Where described, initial clinical improvement was noted within days to weeks after initiating chelation, but complete resolution of symptoms, particularly neurological sequelae, often extended over several months. Even among patients who respond well to treatment, sudden death due to mercury-induced cardiovascular complications may occur (45), highlighting the importance of monitoring mercury-related secondary organ damage. As this study focused primarily on the treatment of surviving patients, the sample size of fatal cases was limited. Further research is needed to explore the mechanisms and preventive strategies associated with mercury-related mortality.

In summary, future strategies for the prevention and treatment of mercury poisoning should include the following targeted measures: enhancing public education to emphasize proper handling of household mercury spills and raising awareness of the health risks associated with mercury-containing cosmetics and folk remedies; promoting the adoption of mercury-free alternatives and gradually phasing out mercury-based medical devices such as thermometers and sphygmomanometers; strengthening occupational health regulations, particularly regarding safety supervision in industries such as artisanal gold mining and metallurgy; and implementing a mandatory detailed inquiry into potential mercury exposure during the evaluation of patients with multi-system involvement of unknown etiology.

### Limitations

Several important limitations of this study must be acknowledged. First, the review was limited to English-language publications, which may have resulted in the omission of relevant data from non-English sources. Furthermore, all 80 included studies were case reports or case series. Although some cross-sectional studies were identified during the search (104), they could not be incorporated due to insufficient clinical details, underscoring the overall scarcity of high-quality, systematic data in this field. The inherent rarity of mercury poisoning largely precludes large-scale prospective studies, making retrospective case synthesis a necessary but suboptimal alternative.

The retrospective and narrative nature of these sources introduces risks of publication bias, as unusual or severe outcomes are more likely to be reported. The generally small sample sizes and absence of control groups further limit the generalizability of the findings. Additionally, the pooled statistical analyses combined data from heterogeneous reports with variations in interventions and outcome measures, complicating direct comparisons and introducing potential heterogeneity.

Another notable limitation is the underrepresentation of certain poisoning types. A systematic search identified only few well-documented cases report of methylmercury poisoning (18, 80). This gap limits the comprehensiveness of the clinical spectrum examined (105). Moreover, asymptomatic or mild poisoning cases are likely underrepresented due to underreporting, and the lack of a universally accepted definition of mercury poisoning adds further inconsistency to case identification and interpretation. Future accumulation of more cases may allow for a more robust meta-analysis and better characterization of mercury poisoning across its full range of manifestations.

## Conclusion

In summary, mercury poisoning remains a major and continually evolving public health issue, manifested through diverse exposure sources and varied clinical presentations. There is still a lack of universal diagnostic criteria, and diagnosis continues to rely on clinical experience and biomarker evidence. Although supportive care and chelation therapy form the basis of treatment, their efficacy is not yet fully established. This study highlights the need to enhance public awareness of common exposure risks, strengthen regulatory and therapeutic measures, and conduct larger-scale clinical research to establish standardized management guidelines.

## Data Availability

The original contributions presented in the study are included in the article/Supplementary material, further inquiries can be directed to the corresponding author.
