# Supplementary material for: Clinical characteristics, management, and outcomes of diseases caused by mercury overexposure: a systematic review of case reports and case series
Source: Front Public Health. 2026 Feb 2;14:1750332. doi: 10.3389/fpubh.2026.1750332 (PMC12907422; doi:10.3389/fpubh.2026.1750332)
Supplement: Supplementary file 1 [file Table_1.DOCX]

Supplementary Table 1 Basic Information of the Case Reports

| Literature information | | | Patient information | | | | |
| --- | --- | --- | --- | --- | --- | --- | --- |
| The First Author | Year | Country | Gender | Age | Source of exposure | Chelation therapy | Outcome |
| I. D. P. Wootton(1) | 1957 | England | M | 3m | Medical/iatrogenic exposure | Yes | Rehabilitation |
| Ching Tseng Teng(2) | 1959 | America | F | 30m | Domestic/environmental exposure | No | Death |
|  |  |  | M | 20m | Domestic/environmental exposure | No | Death |
|  |  |  | F | 4m | Domestic/environmental exposure | No | Death |
|  |  |  | F | 19y | Domestic/environmental exposure | No | Sequelae |
| Aziz A. Rahimo(3) | 1960 | America | M | 56y | Medical/iatrogenic exposure | No | Rehabilitation |
| Carl G. Becker(4) | 1962 | America | M | 50y | Medical/iatrogenic exposure | No | Rehabilitation |
|  |  |  | F | 46y | Medical/iatrogenic exposure | Yes | Poor Prognosis |
|  |  |  | F | 59y | Medical/iatrogenic exposure | No | Rehabilitation |
|  |  |  | M | 40y | Occupational exposure | Yes | Poor Prognosis |
|  |  |  | F | 66y | Medical/iatrogenic exposure | No | Rehabilitation |
| John P. Royle(5) | 1964 | Australia | F | 65y | Medical/iatrogenic exposure | Yes | Rehabilitation |
| Gloria O. Schrager(6) | 1964 | America | M | 2y | Occupational exposure | No | Rehabilitation |
| J. F. Alexander(7) | 1971 | Canada | F | 8y | Domestic/environmental exposure | Yes | Poor Prognosis |
| Paul E. Pierce(8) | 1972 | America | F | 8y | Domestic/environmental exposure | Yes | Sequelae |
|  |  |  | M | 13y | Domestic/environmental exposure | Yes | Basic rehabilitation |
|  |  |  | F | 20y | Domestic/environmental exposure | Yes | Poor Prognosis |
| Eleanor A. Magill(9) | 1975 | England | M | 20m | Domestic/environmental exposure | Yes | Basic rehabilitation |
|  |  |  | M | 10m | Medical/iatrogenic exposure | No | Rehabilitation |
| Francis L. Weber(10) | 1979 | Switzerland | F | 24y | Occupational exposure | Yes | Sequelae |
| Ian S. Symington(11) | 1980 | England | M | 44y | Occupational exposure | Yes | Rehabilitation |
|  |  |  | M | 50y | Occupational exposure | Yes | Rehabilitation |
| Maria E. Moutinho(12) | 1981 | America | F | 7m | Domestic/environmental exposure | Yes | Death |
| E.R. Samuels(13) | 1982 | Canada | M | 19m | Domestic/environmental exposure | Yes | Rehabilitation |
| Kenneth M. Jaffe(14) | 1983 | America | F | 8m | Domestic/environmental exposure | No | Basic rehabilitation |
| T. Stack(15) | 1983 | England | M | 23m | Domestic/environmental exposure | Yes | Rehabilitation |
| Trevor Laundy(16) | 1984 | England | F | 23y | Medical/iatrogenic exposure | No | Death |
| JoAnn Rohyans(17) | 1984 | America | F | 18m | Medical/iatrogenic exposure | Yes | Death |
| D. P. Worth(18) | 1984 | England | M | 29y | Medical/iatrogenic exposure | Yes | Rehabilitation |
| Ruth Lilis(19) | 1985 | America | M | 31y | Occupational exposure | Yes | Sequelae |
| M. Bourgeois(20) | 1986 | Belgium | M | 40y | Medical/iatrogenic exposure | Yes | Poor Prognosis |
|  |  |  | F | / | Medical/iatrogenic exposure | Yes | Basic rehabilitation |
| James R. Campbell(21) | 1986 | America | M | 23y | Occupational exposure | Yes | Rehabilitation |
|  |  |  | M | 22y | Occupational exposure | Yes | Rehabilitation |
| Mark Levin(22) | 1988 | America | M | 19y | Domestic/environmental exposure | Yes | Rehabilitation |
|  |  |  | M | 33y | Domestic/environmental exposure | Yes | Loss to follow-up |
|  |  |  | M | 54y | Domestic/environmental exposure | Yes | Basic rehabilitation |
| Delwyn J Dyall-Smith(23) | 1990 | Australia | F | 42y | Cosmetic exposure | No | Sequelae |
| Bradley Rowens(24) | 1991 | America | M | 41y | Domestic/environmental exposure | Yes | Death |
|  |  |  | F | 40y | Domestic/environmental exposure | Yes | Death |
|  |  |  | F | 88y | Domestic/environmental exposure | Yes | Death |
|  |  |  | M | 69y | Domestic/environmental exposure | Yes | Death |
| Gwen E. Fagala(25) | 1992 | America | F | 12y | Domestic/environmental exposure | Yes | Loss to follow-up |
| Joyce G. Schwartz(26) | 1992 | America | M | 3.5y | Domestic/environmental exposure | Yes | Loss to follow-up |
|  |  |  | M | 5y | Domestic/environmental exposure | Yes | Loss to follow-up |
|  |  |  | M | / | Domestic/environmental exposure | Yes | Loss to follow-up |
|  |  |  | F | / | Domestic/environmental exposure | Yes | Loss to follow-up |
| Banani Bhattacharya(27) | 1997 | India | F | 5m | Domestic/environmental exposure | No | Rehabilitation |
| Dirk Deleu(28) | 1998 | Oman | M | 36y | Medical/iatrogenic exposure | Yes | Sequelae |
| Hong Euy Lim(29) | 1998 | Korea | M | 72y | Folk remedy exposure | Yes | Sequelae |
| A. C. Rennie(30) | 1999 | England | M | 9y | Domestic/environmental exposure | Yes | Loss to follow-up |
| Albert M. Li(31) | 2000 | China | M | 5y | Medical/iatrogenic exposure | No | Rehabilitation |
| Michael T. Solis(32) | 2000 | America | F | 45d | Domestic/environmental exposure | Yes | Rehabilitation |
|  |  |  | M | 13m | Domestic/environmental exposure | Yes | Death |
|  |  |  | F | 38y | Domestic/environmental exposure | Yes | Death |
|  |  |  | M | 58y | Domestic/environmental exposure | No | Sequelae |
|  |  |  | / | 3y | Domestic/environmental exposure | Yes | Rehabilitation |
|  |  |  | / | 7y | Domestic/environmental exposure | Yes | Rehabilitation |
|  |  |  | / | 10y | Domestic/environmental exposure | Yes | Rehabilitation |
|  |  |  | / | 14y | Domestic/environmental exposure | Yes | Rehabilitation |
| Debra Cherry(33) | 2002 | America | F | 3y | Domestic/environmental exposure | Yes | Rehabilitation |
| D. Pelclová(34) | 2002 | Czech Republic | M | 21y | Medical/iatrogenic exposure | Yes | Basic rehabilitation |
| Mustafa Koyun(35) | 2004 | Turkey | M | 13y | Domestic/environmental exposure | Yes | Death |
|  |  |  | M | 16y | Domestic/environmental exposure | Yes | Basic rehabilitation |
|  |  |  | F | 11y | Domestic/environmental exposure | Yes | Rehabilitation |
| HL Tang(36) | 2006 | China | F | 34y | Cosmetic exposure | Yes | Rehabilitation |
| Yael Michaeli-Yossef(37) | 2007 | Israel | F | 2y | / | Yes | Rehabilitation |
| J. M. Setz(38) | 2008 | Netherlands | F | 11y | Domestic/environmental exposure | Yes | Rehabilitation |
| Carline Koh(39) | 2009 | China | M | 11m | Medical/iatrogenic exposure | Yes | Rehabilitation |
| Sezgin Sarikaya(40) | 2009 | Turkey | F | 36y | Domestic/environmental exposure | Yes | Rehabilitation |
| Nilgun Erkek(41) | 2010 | Turkey | F | 10y | / | Yes | Basic rehabilitation |
| Sanjay Verma(42) | 2010 | India | M | 2y | Domestic/environmental exposure | Yes | Rehabilitation |
| Cahide Yilmaz(43) | 2010 | Turkey | M | 7y | Domestic/environmental exposure | Yes | Rehabilitation |
|  |  |  | F | 13y | Domestic/environmental exposure | Yes | Rehabilitation |
| S. Al-Sinani(44) | 2011 | Oman | F | 12y | Medical/iatrogenic exposure | Yes | Death |
| Hasan Tezer(45) | 2011 | Turkey | F | 12y | Domestic/environmental exposure | Yes | Sequelae |
|  |  |  | M | 11y | Domestic/environmental exposure | Yes | Rehabilitation |
|  |  |  | F | 16y | Domestic/environmental exposure | Yes | Sequelae |
| Tarek Alhamad(46) | 2012 | America | M | 36y | Occupational exposure | Yes | Death |
| Elizabeth H. Brannan(47) | 2012 | America | F | 3y | Domestic/environmental exposure | Yes | Rehabilitation |
| Serife Gul Oz(48) | 2012 | Turkey | F | 42y | Domestic/environmental exposure | Yes | Rehabilitation |
|  |  |  | M | 19y | Domestic/environmental exposure | Yes | Rehabilitation |
|  |  |  | M | 20y | Domestic/environmental exposure | Yes | Sequelae |
|  |  |  | M | 49y | Domestic/environmental exposure | No | Rehabilitation |
| Jessica J. Mercer(49) | 2012 | America | F | 3y | Domestic/environmental exposure | Yes | Rehabilitation |
| Federico A. Silva Sieger(50) | 2012 | Colombia | M | 50y | Occupational exposure | Yes | Rehabilitation |
| Rahşan Yıldırım(51) | 2012 | Turkey | F | 54y | Domestic/environmental exposure | Yes | Death |
|  |  |  | M | 29y | Domestic/environmental exposure | Yes | Basic rehabilitation |
|  |  |  | F | 22y | Domestic/environmental exposure | Yes | Basic rehabilitation |
|  |  |  | F | 20y | Domestic/environmental exposure | Yes | Rehabilitation |
|  |  |  | F | 23y | Domestic/environmental exposure | Yes | Rehabilitation |
| Mohamed Saleem(52) | 2013 | Australia | M | 62y | Domestic/environmental exposure | Yes | Rehabilitation |
| K. Sathe(53) | 2013 | India | F | 2y | Medical/iatrogenic exposure | Yes | Rehabilitation |
| Gonul Cicek-Senturk(54) | 2014 | Turkey | F | 52y | Domestic/environmental exposure | Yes | Rehabilitation |
| Xuebing Huang(55) | 2014 | China | M | 42y | Medical/iatrogenic exposure | Yes | Basic rehabilitation |
| Chhaya Divecha(56) | 2015 | India | M | 2y | Domestic/environmental exposure | Yes | Death |
| Uğur Nadir Karakulak(57) | 2015 | Turkey | F | 32y | Domestic/environmental exposure | Yes | Basic rehabilitation |
| Tingting Mo(58) | 2016 | China | F | 35y | Folk remedy exposure | Yes | Basic rehabilitation |
| Radu Ciprian Tincu(59) | 2016 | Romania | F | 28y | Domestic/environmental exposure | No | Rehabilitation |
| M Vahabzadeh(60) | 2016 | Iran | M | 30y | Occupational exposure | Yes | Rehabilitation |
|  |  |  | M | 20y | Occupational exposure | Yes | Basic rehabilitation |
|  |  |  | M | 53y | Occupational exposure | Yes | Rehabilitation |
| George Bazoukis(61) | 2017 | Greece | M | 27y | Occupational exposure | Yes | Rehabilitation |
| Matthew Carter(62) | 2017 | America | M | 12y | Domestic/environmental exposure | Yes | Rehabilitation |
| Zhenyan Gao(63) | 2017 | China | F | 3m | Domestic/environmental exposure | Yes | Rehabilitation |
| Dongmin Kim(64) | 2017 | Korea | F | 43y | Domestic/environmental exposure | No | Rehabilitation |
| Ilka Kleffner(65) | 2017 | Germany | M | 23y | / | Yes | Sequelae |
| Alexandre Malek(66) | 2017 | Lebanon | M | 28y | Occupational exposure | No | Rehabilitation |
| Oluwadamilola Onwuzuligbo(67) | 2018 | America | M | 14y | Domestic/environmental exposure | Yes | Basic rehabilitation |
| Amelia Geraldine Peregrina-Chavez(68) | 2018 | Mexico | F | 52y | Domestic/environmental exposure | Yes | Sequelae |
| Olivia L. Kamensky(69) | 2019 | America | F | 32y | Domestic/environmental exposure | Yes | Loss to follow-up |
| Anita Mudan(70) | 2019 | America | F | 47y | Cosmetic exposure | Yes | Sequelae |
| Jin Yan(71) | 2019 | China | M | 42y | Occupational exposure | Yes | Poor Prognosis |
|  |  |  | F | 10y | Domestic/environmental exposure | Yes | Rehabilitation |
| Amy C. Young(72) | 2020 | America | M | 14y | Domestic/environmental exposure | Yes | Rehabilitation |
|  |  |  | F | 9y | Domestic/environmental exposure | Yes | Rehabilitation |
|  |  |  | F | 11y | Domestic/environmental exposure | Yes | Basic rehabilitation |
| Jan Magdalan(73) | 2021 | Poland | M | 21y | Domestic/environmental exposure | Yes | Basic rehabilitation |
| Stefan Rakete(74) | 2021 | Germany | M | 4y | Cosmetic exposure | Yes | Rehabilitation |
| Enrong Ran(75) | 2021 | China | F | 56y | Medical/iatrogenic exposure | Yes | Rehabilitation |
| Zhongying Wang(76) | 2021 | China | F | 45y | Cosmetic exposure | Yes | Basic rehabilitation |
| Chen Yawei(77) | 2021 | China | F | 44y | Cosmetic exposure | Yes | Basic rehabilitation |
| Khoa Nguyen-Dang(78) | 2024 | Vietnam | F | 47y | Domestic/environmental exposure | Yes | Rehabilitation |
| Ahmed Moustafa Elmenshawy(79) | 2025 | Egypt | / | 3.5y | Occupational exposure | Yes | Rehabilitation |
| Huixia Ji(80) | 2025 | China | M | 33y | Cosmetic exposure | Yes | Poor Prognosis |
|  |  |  | F | 30y | Cosmetic exposure | Yes | Rehabilitation |

Notes: M, Male; F, Female; y, years; m, months; d, days.

Supplementary Table 2 JBI Critical Appraisal Checklist for case reports

|  | The First Author | Year | 1.Were patient’s demographic characteristics clearly described? | 2.Was the patient’s history clearly described and presented as a timeline? | 3.Was the current clinical condition of the patient on presentation clearly described? | 4.Were diagnostic tests or assessment methods and the results clearly described? | 5.Was the intervention(s) or treatment procedure(s) clearly described? | 6.Was the post-intervention clinical condition clearly described? | 7.Were adverse events (harms) or unanticipated events identified and described? | 8.Does the case report provide takeaway lessons? |
| --- | --- | --- | --- | --- | --- | --- | --- | --- | --- | --- |
| 1 | I. D. P. Wootton(1) | 1957 | √ | √ | √ | √ | √ | √ | × | √ |
| 2 | Ching Tseng Teng(2) | 1959 | √ | √ | √ | √ | × | √ | √ | √ |
| 3 | Aziz A. Rahimo(3) | 1960 | √ | √ | √ | × | √ | √ | × | √ |
| 4 | Carl G. Becker(4) | 1962 | √ | √ | √ | × | √ | √ | × | √ |
| 5 | John P. Royle(5) | 1964 | √ | √ | √ | √ | √ | √ | √ | √ |
| 6 | Gloria O. Schrager(6) | 1964 | √ | √ | √ | √ | √ | √ | √ | √ |
| 7 | J. F. Alexander(7) | 1971 | √ | √ | √ | √ | √ | √ | √ | √ |
| 8 | Paul E. Pierce(8) | 1972 | √ | √ | √ | √ | √ | √ | √ | √ |
| 9 | Eleanor A. Magill(9) | 1975 | √ | √ | √ | √ | √ | √ | × | √ |
| 10 | Francis L. Weber(10) | 1979 | √ | √ | √ | √ | √ | √ | × | √ |
| 11 | Ian S. Symington(11) | 1980 | √ | √ | √ | √ | √ | √ | × | √ |
| 12 | Maria E. Moutinho(12) | 1981 | √ | √ | √ | √ | × | √ | √ | √ |
| 13 | E.R. Samuels(13) | 1982 | √ | √ | √ | √ | √ | √ | × | √ |
| 14 | Kenneth M. Jaffe(14) | 1983 | √ | √ | √ | √ | √ | √ | √ | √ |
| 15 | T. Stack(15) | 1983 | √ | √ | √ | √ | √ | √ | × | √ |
| 16 | Trevor Laundy(16) | 1984 | √ | √ | √ | √ | √ | √ | √ | √ |
| 17 | JoAnn Rohyans(17) | 1984 | √ | √ | √ | √ | √ | √ | √ | √ |
| 18 | D. P. Worth(18) | 1984 | √ | √ | √ | √ | √ | √ | √ | √ |
| 19 | Ruth Lilis(19) | 1985 | √ | √ | √ | √ | √ | √ | √ | √ |
| 20 | M. Bourgeois(20) | 1986 | √ | × | √ | √ | √ | √ | × | √ |
| 21 | James R. Campbell(21) | 1986 | √ | × | √ | √ | √ | √ | × | √ |
| 22 | Mark Levin(22) | 1988 | √ | √ | √ | √ | √ | × | × | √ |
| 23 | Delwyn J Dyall-Smith(23) | 1990 | √ | √ | √ | √ | × | √ | × | √ |
| 24 | Bradley Rowens(24) | 1991 | √ | √ | √ | √ | √ | √ | √ | √ |
| 25 | Gwen E. Fagala(25) | 1992 | √ | √ | √ | √ | √ | × | √ | √ |
| 26 | Joyce G. Schwartz(26) | 1992 | √ | √ | √ | √ | √ | × | √ | √ |
| 27 | Banani Bhattacharya(27) | 1997 | √ | √ | √ | × | √ | √ | √ | √ |
| 28 | Dirk Deleu(28) | 1998 | √ | √ | √ | √ | √ | √ | √ | √ |
| 29 | Hong Euy Lim(29) | 1998 | √ | √ | √ | √ | √ | √ | √ | √ |
| 30 | A. C. Rennie(30) | 1999 | √ | √ | √ | √ | √ | √ | √ | √ |
| 31 | Albert M. Li(31) | 2000 | √ | √ | √ | √ | √ | √ | √ | √ |
| 32 | Michael T. Solis(32) | 2000 | √ | √ | √ | √ | √ | √ | √ | √ |
| 33 | Debra Cherry(33) | 2002 | √ | √ | √ | √ | √ | √ | √ | √ |
| 34 | D. Pelclová(34) | 2002 | √ | √ | √ | √ | √ | √ | √ | √ |
| 35 | Mustafa Koyun(35) | 2004 | √ | √ | √ | √ | √ | √ | √ | √ |
| 36 | HL Tang(36) | 2006 | √ | √ | √ | √ | √ | √ | × | √ |
| 37 | Yael Michaeli-Yossef(37) | 2007 | √ | √ | √ | √ | √ | √ | √ | √ |
| 38 | J. M. Setz(38) | 2008 | √ | √ | √ | √ | √ | √ | × | √ |
| 39 | Carline Koh(39) | 2009 | √ | √ | √ | √ | √ | √ | × | √ |
| 40 | Sezgin Sarikaya(40) | 2009 | √ | √ | √ | × | √ | √ | √ | √ |
| 41 | Nilgun Erkek(41) | 2010 | √ | √ | √ | √ | √ | √ | × | √ |
| 42 | Sanjay Verma(42) | 2010 | √ | √ | √ | √ | √ | √ | × | √ |
| 43 | Cahide Yilmaz(43) | 2010 | √ | √ | √ | √ | √ | √ | √ | √ |
| 44 | S. Al-Sinani(44) | 2011 | √ | √ | √ | √ | √ | √ | √ | √ |
| 45 | Hasan Tezer(45) | 2011 | √ | √ | √ | √ | √ | √ | × | √ |
| 46 | Tarek Alhamad(46) | 2012 | √ | √ | √ | √ | √ | √ | √ | √ |
| 47 | Elizabeth H. Brannan(47) | 2012 | √ | √ | √ | √ | √ | √ | × | √ |
| 48 | Serife Gul Oz(48) | 2012 | √ | √ | √ | √ | √ | √ | × | √ |
| 49 | Jessica J. Mercer(49) | 2012 | √ | √ | √ | √ | × | √ | × | √ |
| 50 | Federico A. Silva Sieger(50) | 2012 | √ | √ | √ | √ | × | × | × | √ |
| 51 | Rahşan Yıldırım(51) | 2012 | √ | √ | √ | √ | × | √ | × | √ |
| 52 | Mohamed Saleem(52) | 2013 | √ | √ | √ | √ | √ | √ | √ | √ |
| 53 | K. Sathe(53) | 2013 | √ | √ | √ | √ | √ | √ | √ | √ |
| 54 | Gonul Cicek-Senturk(54) | 2014 | √ | √ | √ | √ | √ | √ | × | √ |
| 55 | Xuebing Huang(55) | 2014 | √ | √ | √ | √ | √ | √ | × | √ |
| 56 | Chhaya Divecha(56) | 2015 | √ | √ | √ | √ | √ | √ | × | √ |
| 57 | Uğur Nadir Karakulak(57) | 2015 | √ | √ | √ | √ | √ | √ | × | √ |
| 58 | Tingting Mo(58) | 2016 | √ | √ | √ | √ | √ | √ | × | √ |
| 59 | Radu Ciprian Tincu(59) | 2016 | √ | √ | √ | √ | √ | √ | × | √ |
| 60 | M Vahabzadeh(60) | 2016 | √ | √ | √ | √ | √ | √ | × | √ |
| 61 | George Bazoukis(61) | 2017 | √ | √ | √ | √ | √ | × | × | √ |
| 62 | Matthew Carter(62) | 2017 | √ | × | √ | √ | √ | √ | × | √ |
| 63 | Zhenyan Gao(63) | 2017 | √ | √ | √ | √ | × | √ | × | √ |
| 64 | Dongmin Kim(64) | 2017 | √ | √ | √ | √ | √ | √ | × | √ |
| 65 | Ilka Kleffner(65) | 2017 | √ | √ | √ | √ | √ | √ | × | √ |
| 66 | Alexandre Malek(66) | 2017 | √ | √ | √ | √ | √ | √ | × | √ |
| 67 | Oluwadamilola Onwuzuligbo(67) | 2018 | √ | √ | √ | √ | √ | √ | √ | √ |
| 68 | Amelia Geraldine Peregrina-Chavez(68) | 2018 | √ | √ | √ | √ | √ | √ | √ | √ |
| 69 | Olivia L. Kamensky(69) | 2019 | √ | √ | √ | √ | √ | √ | × | √ |
| 70 | Anita Mudan(70) | 2019 | √ | √ | √ | √ | √ | √ | √ | √ |
| 71 | Jin Yan(71) | 2019 | √ | √ | √ | √ | √ | √ | √ | √ |
| 72 | Amy C. Young(72) | 2020 | √ | √ | √ | √ | √ | √ | × | √ |
| 73 | Jan Magdalan(73) | 2021 | √ | √ | √ | √ | √ | √ | × | √ |
| 74 | Stefan Rakete(74) | 2021 | √ | √ | √ | √ | √ | √ | × | √ |
| 75 | Enrong Ran(75) | 2021 | √ | √ | √ | √ | √ | √ | × | √ |
| 76 | Zhongying Wang(76) | 2021 | √ | √ | √ | √ | √ | √ | √ | √ |
| 77 | Chen Yawei(77) | 2021 | √ | √ | √ | √ | √ | √ | × | √ |
| 78 | Khoa Nguyen-Dang(78) | 2024 | √ | √ | √ | √ | √ | √ | × | √ |
| 79 | Ahmed Moustafa Elmenshawy(79) | 2025 | × | √ | √ | √ | √ | √ | √ | √ |
| 80 | Huixia Ji(80) | 2025 | √ | √ | √ | √ | √ | √ | × | √ |

Note: JBI, Joanna Briggs Institute.

**References:**

1. Wootton ID, Giddings AG. Acute mercury poisoning in infancy; report of a case. *Lancet*. (1957) 273: 1038-1039. doi:10.1016/S0140-6736(57)92155-4.

2. Teng CT, Brennan JC. Acute Mercury Vapor Poisoning. *Radiology*. (1959) 73: 354-361. doi:10.1148/73.3.354.

3. Rahimo AA, Shimasaki WW. Mercurial necrosis of the cheek. *Oral Surg Oral Med Oral Pathol*. (1960) 13: 54-58. doi:10.1016/0030-4220(60)90392-3.

4. Becker CG. Nephrotic Syndrome After Contact with Mercury. *Arch Intern Med*. (1962) 110: 178-186. doi:10.1001/archinte.1962.03620200038008.

5. Royle JP. A Case of Mercury Poisoning Following the Use of Perchloride of Mercury. *Aust N Z J Surg*. (2008) 34: 71-72. doi:10.1111/j.1445-2197.1964.tb03990.x.

6. Schrager GO. Acute mercury poisoning in a child following contact with marine antifouling paint. *J Pediatr*. (1964) 65: 780-782. doi:10.1016/s0022-3476(64)80166-9.

7. Alexander JF, Rosario R. A case of mercury poisoning: acrodynia in a child of 8. *Can Med Assoc J*. (1971) 104: 929-930.

8. Pierce PE, Thompson JF, Likosky WH, Nickey LN, Barthel WF, Hinman AR. Alkyl mercury poisoning in humans. Report of an outbreak. *JAMA*. (1972) 220: 1439-1442.

9. Magill EA. Mercury Poisoning in Children (A report of two patients). *Ulster Med J*. (1975) 44: 166-170.

10. Weber FL, Babel J. Corneal trauma from projection of metallic mercury into the eyes. *Arch Ophthalmol*. (1979) 97: 1116-1120. doi:10.1001/archopht.1979.01020010570013.

11. Symington IS, Cross JD, Dale IM, Lenihan JM. Mercury poisoning in dentists. *J Soc Occup Med*. (1980) 30: 37-39.

12. Moutinho ME, Tompkins AL, Rowland TW. Acute mercury vapor poisoning. Fatality in an infant. *Am J Dis Child*. (1981) 135: 42-44. doi:10.1001/archpedi.1981.02130250030010.

13. Samuels ER, Heick HMC, McLaine PN, Farant JP. A case of accidental inorganic mercury poisoning. *J Anal Toxicol*. (1982) 6: 120-122. doi:10.1093/jat/6.3.120.

14. Jaffe KM, Shurtleff DB, Robertson WO. Survival after acute mercury vapor poisoning. Role of intensive supportive care. *Am J Dis Child*. (1983) 137: 749-751. doi:10.1001/archpedi.1983.02140340033008.

15. Stack T, Bissenden JG, Hoffman G, Yeoman WB. Mercuric chloride poisoning in a 23 month old child. *BMJ*. (1983) 287: 1513. doi:10.1136/bmj.287.6404.1513.

16. Laundy T, Adam AE, Kershaw JB, Rainford DJ. Deaths after peritoneal lavage with mercuric chloride solutions: case report and review of the literature. *BMJ*. (1984) 289: 96-98. doi:10.1136/bmj.289.6437.96.

17. Rohyans J, Walson PD, Wood GA, MacDonald WA. Mercury toxicity following merthiolate ear irrigations. *J Pediatr*. (1984) 104: 311-313. doi:10.1016/S0022-3476(84)81021-5.

18. Worth DP, Davison AM, Lewins AM. Haemodialysis and charcoal haemoperfusion in acute inorganic mercury poisoning. *Postgrad Med J*. (1984) 60: 636-638. doi:10.1136/PGMJ.60.707.636.

19. Lilis R, Miller A, Lerman Y. Acute Mercury Poisoning with Severe Chronic Pulmonary Manifestations. *Chest*. (1985) 88: 306-309. doi:10.1378/chest.88.2.306.

20. Bourgeois M, Dooms-Goossens A, Knockaert D, Sprengers D, Van Boven M, Van Tittelboom T. Mercury Intoxication after Topical Application of a Metallic Mercury Ointment. *Dermatology*. (1986) 172: 48-51. doi:10.1159/000249292.

21. Campbell JR. The therapeutic use of 2,3-dimercaptopropane-1-sulfonate in two cases of inorganic mercury poisoning. *JAMA: The Journal of the American Medical Association*. (1986) 256: 3127-3130. doi:10.1001/jama.1986.03380220093029.

22. Levin M, Jacobs J, Polos PG. Acute Mercury Poisoning and Mercurial Pneumonitis from Gold Ore Purification. *Chest*. (1988) 94: 554-556. doi:10.1378/chest.94.3.554.

23. Dyall‐Smith DJ, Scurry JP. Mercury pigmentation and high mercury levels from the use of a cosmetic cream. *Med J Aust*. (1990) 153: 409-415. doi:10.5694/j.1326-5377.1990.tb125501.x.

24. Rowens B, Guerrero-Betancourt D, Gottlieb CA, Boyes RJ, Eichenhorn MS. Respiratory Failure and Death Following Acute Inhalation of Mercury Vapor. *Chest*. (1991) 99: 185-190. doi:10.1378/chest.99.1.185.

25. Fagala GE, Wigg CL. Psychiatric manifestations of mercury poisoning. *J Am Acad Child Adolesc Psychiatry*. (1992) 31: 306-311. doi:10.1097/00004583-199203000-00019.

26. Schwartz JG, Snider TE, Montiel MM. Toxicity of a family from vacuumed mercury. *Am J Emerg Med*. (1992) 10: 258-261. doi:10.1016/0735-6757(92)90221-I.

27. Bhattacharya B, Banerjee S, Singhi S. Acute mercury vapour poisoning in an infant. *Ann Trop Paediatr*. (2016) 17: 57-60. doi:10.1080/02724936.1997.11747864.

28. Deleu D, Hanssens Y, Al-Salmy HS, Hastie I. Peripheral Polyneuropathy Due to Chronic Use of Topical Ammoniated Mercury. *J Toxicol Clin Toxicol*. (2009) 36: 233-237. doi:10.3109/15563659809028945.

29. Lim HE, Shim JJ, Lee SY, Lee SH, Jo JY, In KH, et al. Mercury inhalation poisoning and acute lung injury. *Korean J Inter Med*. (1998) 13: 127-130. doi:10.3904/kjim.1998.13.2.127.

30. Rennie AC, McGregor-Schuerman M, Dale IM, Robinson C, McWilliam R. Mercury poisoning after spillage at home from a sphygmomanometer on loan from hospital. *Br Med J*. (1999) 319: 366-367. doi:10.1136/bmj.319.7206.366.

31. Li AM. Short report: Mercury intoxication presenting with tics. *Arch Dis Child*. (2000) 83: 174-175. doi:10.1136/adc.83.2.174.

32. Solis MT, Yuen E, Cortez PS, Goebel PJ. Family poisoned by mercury vapor inhalation. *Am J Emerg Med*. (2000) 18: 599-602. doi:10.1053/ajem.2000.4006.

33. Cherry D, Lowry L, Velez L, Cotrell C, Keyes DC. Elemental mercury poisoning in a family of seven. *Fam Community Health*. (2002) 24: 1-8. doi:10.1097/00003727-200201000-00003.

34. Pelclova D, Lukas E, Urban P, Preiss J, Rysava R, Lebenhart P, et al. Mercury intoxication from skin ointment containing mercuric ammonium chloride. *Int Arch Occup Environ Health*. (2002) 75: 54-59. doi:10.1007/s00420-002-0349-x.

35. Koyun M, Akman S, Güven AG. Mercury intoxication resulting from school barometers in three unrelated adolescents. *Eur J Pediatr*. (2004) 163: 131-134. doi:10.1007/s00431-003-1389-2.

36. Tang HL, Chu KH, Mak YF, Lee W, Cheuk A, Yim KF, et al. Minimal change disease following exposure to mercury-containing skin lightening cream. *Hong Kong Med J*. (2006) 12: 316-318.

37. Michaeli-Yossef Y, Berkovitch M, Goldman M. Mercury intoxication in a 2-year-old girl: a diagnostic challenge for the physician. *Pediatr Nephrol*. (2007) 22: 903-906. doi:10.1007/s00467-007-0430-5.

38. Setz JM, van der Linde AAA, Gerrits GPJM, Meulstee J. EEG Findings in an Eleven-Year-Old Girl with Mercury Intoxication. *Clin EEG Neurosci*. (2008) 39: 210-213. doi:10.1177/155005940803900412.

39. Koh C, Kwong KL, Wong SN. Mercury poisoning: A rare but treatable cause of failure to thrive and developmental regression in an infant. *Hong Kong Med J*. (2009) 15: 61-64.

40. Sarikaya S, Karcioglu O, Ay D, Cetin A, Aktas C, Serinken M. Acute mercury poisoning: a case report. *BMC Emerg Med*. (2010) 10: 7. doi:10.1186/1471-227x-10-7.

41. Erkek N, Senel S, Sarac A, Ertan U, Karacan CD. Being alive after a severe inorganic mercury intoxication. *Eur J Pediatr*. (2010) 169: 625-628. doi:10.1007/s00431-009-1073-2.

42. Verma S, Kumar R, Khadwal A, Singhi S. Accidental Inorganic Mercury Chloride Poisoning in a 2-Year Old Child. *Indian J Pediatr*. (2010) 77: 1153-1155. doi:10.1007/s12098-010-0143-9.

43. Okur M, Yilmaz C, Geylani H, Caksen H, Tuncer O, Atas B. Chronic mercury poisoning: Report of two siblings. *Indian J Occup Environ Med*. (2010) 14: 17-19. doi:10.4103/0019-5278.64610.

44. Al-Sinani S, Al-Rawas A, Dhawan A. Mercury as a cause of fulminant hepatic failure in a child: Case report and literature review. *Clin Res Hepatol Gastroenterol*. (2011) 35: 580-582. doi:10.1016/j.clinre.2011.06.006.

45. Tezer H, Erkoçoglu M, Kara A, Bayrakci B, Düzova A, Teksam Ö, et al. Household poisoning cases from mercury brought from school. *Eur J Pediatr*. (2011) 170: 397-400. doi:10.1007/s00431-010-1317-1.

46. Alhamad T, Rooney J, Nwosu A, MacCombs J, Kim YS, Shukla V. Lessons learned from a fatal case of mercury intoxication. *Int Urol Nephrol*. (2012) 44: 647-651. doi:10.1007/s11255-010-9896-3.

47. Brannan EH, Su S, Alverson BK. Elemental Mercury Poisoning Presenting as Hypertension in a Young Child. *Pediatr Emerg Care*. (2012) 28: 812-814. doi:10.1097/PEC.0b013e3182628a05.

48. Gul Oz S, Tozlu M, Yalcin SS, Sozen T, Sain Guven G. Mercury vapor inhalation and poisoning of a family. *Inhal Toxicol*. (2012) 24: 652-658. doi:10.3109/08958378.2012.708677.

49. Mercer JJ, Bercovitch L, Muglia JJ. Acrodynia and Hypertension in a Young Girl Secondary to Elemental Mercury Toxicity Acquired in the Home. *Pediatr Dermatol*. (2012) 29: 199-201. doi:10.1111/j.1525-1470.2012.01737.x.

50. Silva Sieger FA, Díaz Silva GA, Ardila GP, García RG. Mercury chronic toxicity might be associated to some cases of hydrocephalus in adult humans? *Med Hypotheses*. (2012) 79: 13-16. doi:10.1016/j.mehy.2012.03.022.

51. Yildirim R, Erdem F, Gundogdu M, Bilen Y, Koca E, Yillikoglu Y, et al. Mercury toxicity-Family case report. *Turk J Hematol*. (2011) 29: 76-79. doi:10.5152/tjh.2011.16.

52. Saleem M, Alfred S, Bahnisch RA, Coates P, Kearney DJ. Mercury poisoning from home gold amalgam extraction. *Med J Aust*. (2013) 199: 125-127. doi:10.5694/mja13.10171.

53. Ali U, Sathe K, Ohri A. Acute renal failure secondary to ingestion of ayurvedic medicine containing mercury. *Indian J Nephrol*. (2013) 23: 301-303. doi:10.4103/0971-4065.114485.

54. Cicek-Senturk G, Altay FA, Ulu-Kilic A, Gurbuz Y, Tutuncu E, Sencan I. Acute mercury poisoning presenting as fever of unknown origin in an adult woman: a case report. *J Med Case Reports*. (2014) 8: 266. doi:10.1186/1752-1947-8-266.

55. Huang XB, Law S, Li D, Yu X, Li B. Mercury Poisoning: A Case of a Complex Neuropsychiatric Illness. *Am J Psychiatry*. (2014) 171: 1253-1256. doi:10.1176/appi.ajp.2013.12101266.

56. Shaikh S, Agrawal M, Deshmukh C, Agarwal S, Divecha C, Tullu M. Acute Respiratory Distress Syndrome Caused by Mercury Inhalation: A Case Report. *J Pediatr Intensive Care*. (2015) 04: 168-170. doi:10.1055/s-0035-1559826.

57. Karakulak UN, Gündüzöz M, Tutkun E, Yilmaz ÖH. An arrhythmic episode after mercury exposure and successful treatment with chelation therapy: A case report. *Anatol J Cardiol*. (2015) 15: 589-590. doi:10.5152/akd.2015.6282.

58. Mo TT, Sun S, Wang YY, Luo D, Peng B, Xia YY. Mercury poisoning caused by Chinese folk prescription (CFP) A case report and analysis of both CFP and quackery. *Medicine*. (2016) 95: e5162. doi:10.1097/MD.0000000000005162.

59. Ţincu RC, Cobilinschi C, Ghiorghiu Z, Macovei RA. Acute mercury poisoning from occult ritual use. *Rom J Anaesth Intensive Care*. (2016) 23: 73-76. doi:10.21454/rjaic.7518.231.mep.

60. Vahabzadeh M, Balali-Mood M. Occupational Metallic Mercury Poisoning in Gilders. *Int J Occup Med Env*. (2016) 7: 116-122. doi:10.15171/ijoem.2016.776.

61. Bazoukis G, Papadatos SS, Michelongona P, Fragkou A, Yalouris A. Assessment and management of elemental mercury poisoning—a case report. *Clin Case Rep*. (2017) 5: 126-129. doi:10.1002/ccr3.811.

62. Carter M, Abdi A, Naz F, Thabet F, Vyas A. A Mercury Toxicity Case Complicated by Hyponatremia and Abnormal Endocrinological Test Results. *Pediatrics*. (2017) 140. doi:10.1542/peds.2016-1402.

63. Gao ZY, Ying XL, Yan J, Wang J, Cai SZ, Yan CH. Acute mercury vapor poisoning in a 3-month-old infant: A case report. *Clin Chim Acta*. (2017) 465: 119-122. doi:10.1016/j.cca.2016.12.019.

64. Kim D, Park JW. Metallic Mercury Injection in the Hand Caused by A Broken Mercury Thermometer: A Case Report. *J Hand Surg-Asian-Pa*. (2017) 22: 519-522. doi:10.1142/s0218810417720376.

65. Kleffner I, Eichler S, Ruck T, Schüngel L, Pfeuffer S, Polzer P, et al. An Enigmatic Case of Acute Mercury Poisoning: Clinical, Immunological Findings and Platelet Function. *Front Neurol*. (2017) 8. doi:10.3389/fneur.2017.00517.

66. Malek A, Aouad K, El khoury R, Halabi-Tawil M, Choucair J. Chronic Mercury Intoxication Masquerading as Systemic Disease: A Case Report and Review of the Literature. *EJCRIM*. (2017) 2: 000632. doi:10.12890/2017_000623.

67. Onwuzuligbo O, Hendricks AR, Hassler J, Domanski K, Goto C, Wolf MTF. Mercury Intoxication as a Rare Cause of Membranous Nephropathy in a Child. *Am J Kidney Dis*. (2018) 72: 601-605. doi:10.1053/j.ajkd.2018.05.013.

68. Peregrina-Chávez A, Ramírez-Galindo M, Chávez-Martínez R, Delahanty-Delgado C, Vazquez-Alaniz F. Full Atrioventricular Block Secondary to Acute Poisoning Mercury: A Case Report. *Int J Environ Res Public Health*. (2018) 15. doi:10.3390/ijerph15040657.

69. Kamensky OL, Horton D, Kingsley DP, Bridges CC. A Case of Accidental Mercury Intoxication. *J Emerg Med*. (2019) 56: 275-278. doi:10.1016/j.jemermed.2018.12.039.

70. Mudan A, Copan L, Wang R, Pugh A, Lebin J, Barreau T, et al. Notes from the Field: Methylmercury Toxicity from a Skin Lightening Cream Obtained from Mexico - California, 2019. *MMWR Morbidity and Mortality Weekly Report*. (2019) 68: 1166-1167. doi:10.15585/mmwr.mm6850a4.

71. Yan J, Pan YJ, Tang ZR, Song YG. Mercury Poisoning Presenting With Hypertension: Report of 2 Cases. *Am J Med*. (2019) 132: 1475-1477. doi:10.1016/j.amjmed.2019.03.050.

72. Young AC, Wax PM, Feng SY, Kleinschmidt KC, Ordonez JE. Acute Elemental Mercury Poisoning Masquerading as Fever and Rash. *J Med Toxicol*. (2020) 16: 470-476. doi:10.1007/s13181-020-00792-6.

73. Magdalan J, Sozanski T, Nowak K, Zawadzki M. Acute intranasal intoxication with mercuric chloride taken accidently instead of cocaine-A case report. *J Forensic Leg Med*. (2021) 78: 1-8. doi:10.1016/j.jflm.2021.102129.

74. Rakete S, Asenbauer E, Böhm S, Leiz S, Peters J, Nowak D, et al. Mercury poisoning of a 4-year-old child by indirect contact to a mercury-containing facial cream: A case report. *SAGE Open Med Case Rep*. (2021) 9: 2050313x211025227. doi:10.1177/2050313x211025227.

75. Ran ER, Wang MH, Yi YX, Feng M, Liu YJ. Mercury poisoning complicated by acquired neuromyotonia syndrome A case report. *Medicine*. (2021) 100: e26910. doi:10.1097/MD.0000000000026910.

76. Wang Z, Fang X. Chronic Mercury Poisoning From Daily Cosmetics: Case Report and Brief Literature Review. *Cureus*. (2021) 13: e19916. doi:10.7759/cureus.19916.

77. Yawei C, Jing S, Wenju S, Yupeng L, Ping Z, Liping H. Mercury as a cause of membranous nephropathy and Guillain–Barre syndrome: case report and literature review. *J Int Med Res*. (2021) 49: 300060521999756. doi:10.1177/0300060521999756.

78. Nguyen-Dang K, Dau-Nguyen AT, Tran-Ngoc N, Duong-Minh N, Dang-Vu T, Nguyen-Ngoc S, et al. Long-term follow-up after acute mercury poisoning-induced pneumonitis following cinnabar heating: A rare case report. *Medicine*. (2024) 103: e41013. doi:10.1097/MD.0000000000041013.

79. Elmenshawy AM, Kholief M, Ghitani SA, El-Banna A, Elkeraie AF, Yassen NA, et al. Successful management after ingestion of a potentially fatal dose of inorganic mercuric chloride: A case report. *Toxicol Rep*. (2025) 14: 101963. doi:10.1016/j.toxrep.2025.101963.

80. Ji HX, Chen Y, Liu DD, Zhou TZ, Tang YH. Diverse clinical manifestations and prognosis in a couple's mercury poisoning caused by skin-lightening creams: two case reports and literature review. *Front Med*. (2025) 11. doi:10.3389/fmed.2024.1511493.
